# More to gain: dietary energy density is related to smoking status in US adults

**DOI:** 10.1186/s12889-018-5248-5

**Published:** 2018-04-04

**Authors:** R. Ross MacLean, Alexandra Cowan, Jacqueline A. Vernarelli

**Affiliations:** 1 VA Connecticut Healthcare System, 950 Campbell Ave, West Haven, CT 06516 USA; 2 Yale University School of Medicine, New Haven, CT 06520 USA; 3 Department of Biology, Fairfield University, 238 Bannow Science Center, Fairfield, CT 06824 USA; 4 Department of Biology, Fairfield University, 216 Bannow Science Center, Fairfield, CT 06824 USA

**Keywords:** NHANES, Smokers, Energy density (ED), CDC, Diet quality

## Abstract

**Background:**

Given the current prevalence of both cigarette use and obesity in the United States, identification of dietary patterns that reduce mortality risk are important public health priorities. The objective of the present study was to evaluate the correlation between cigarette use and dietary energy density, a marker for diet quality, in a population of current smokers, former smokers, and never smokers.

**Methods:**

Data from a nationally representative sample of 5293 adults who participated in the 2013–2014 National Health and Nutrition Examination Surveys (NHANES) were analyzed. Specific survey procedures were used in the analysis to account for sample weights, unequal selection probability, and clustered design when evaluating the association between dietary energy density (ED, energy per weight of food, kcal/g) and current smoking status. Never smokers reported < 100 lifetime cigarettes. Smokers were identified as individuals reporting > 100 lifetime cigarettes and current smoking status was recorded as daily, some days (nondaily), or not at all (former).

**Results:**

A strong linear relationship was observed between smoking pattern and dietary ED in current smokers. Compared to never smokers, daily smokers and nondaily smokers have significantly higher dietary ED (1.79 vs. 2.02 and 1.88, respectively; both *p* < 0.05); demonstrating that any amount of current cigarette consumption is associated with poor diet. Though former smokers had a higher dietary ED than never smokers, this difference still significantly lower than that of current smokers (*p* = 0.002).

**Conclusion:**

These findings suggest that smoking status is associated with poor diet quality. Former smokers had a slightly lower ED value (1.84) than current non-daily smokers (1.89) but a higher value than never smokers (1.79).

## Background

Smoking is the leading cause of preventable mortality and is associated with a variety of chronic illnesses including cardiovascular disease (CVD), cancer, and stroke [1, 2]. Although nearly 7 in 10 adult cigarette smokers want to quit [3], cessation rates remain alarmingly low. For example, the percentage of smokers reporting a serious quit attempt has significantly increased in recent years from 51.2% in 2011 to 55.0% in 2014; however, successful abstinence has remained unchanged at approximately 20% during the same period [4]. Notably, the number of serious quit attempts is well below the 80% objective outlined in Healthy People 2020 [5]. Therefore, additional efforts are needed to increase the number of people making a serious quit attempt and, by proxy, decrease the impact and incidence of chronic medical conditions associated with smoking. Evaluation of modifiable risk factors that are associated with cigarette smoking can help identify targets that are amendable to intervention.

Along with smoking, poor diet is in the top three modifiable risk factors for CVD [6]. Poor diet is a primary determinant of obesity; however, cigarette smoking is negatively associated with obesity, potentially due to the pharmacological effects of nicotine [7]. Despite lower rates of obesity, cigarette smokers have worse diets that are low in essential nutrients compared to nonsmokers and former smokers [8, 9]. A large meta-analytic study comparing smokers (*n* = 35,870) to nonsmokers (*n* = 47,250) across 51 studies revealed that smokers reported greater intakes of energy, total and saturated fat, and cholesterol; while reporting lower intakes of antioxidant vitamins and fiber [10]. Fruits and vegetables are low energy foods that contain high levels of antioxidant vitamins and fiber, so it is not surprising that multiple studies report lower fruit and vegetable consumption among smokers [8, 11–13]. Therefore, a diet quality measure that highlights fruit and vegetable intake and is associated with chronic disease may provide inroads to evaluation and treatment of cigarette smokers.

Dietary energy density (ED, kcal/g) is an established risk factor for obesity and other forms of chronic disease [14, 15]. From a public health perspective, recommendations to consume a diet low in energy density has been recognized by national and international public health organizations, including the *2015 Dietary Guidelines for Americans,* which recommends a low-ED diet as a strategy for prevention of obesity, and as a method of weight control [16]. Diets low in energy density have been characterized as higher in specific low-ED foods, such as fruits and vegetables, and higher in overall diet quality [17–19]. Furthermore, research has suggested that a diet that includes greater consumption of low energy dense foods is associated with a reduction in CVD risk [20, 21].

Diet quality is a comprehensive evaluation of overall dietary intake. Previous studies have demonstrated that dietary ED to be a marker for diet quality in both children and adults, as well as an established risk factor for obesity [17–19, 22] though not when looking specifically at a population of smokers. The relationship between dietary energy density, diet quality, and smoking behaviors is not well understood. To our knowledge, only one study has assessed overall diet quality and smoking status. Alkerwi and colleagues [23] evaluated smoking status and overall diet quality as measured by eight diet quality indices, including overall energy density, using the Observation of Cardiovascular Risk Factors in Luxemburg (ORISCAV-LUX) survey. Notably, the authors divided smoking status into never smokers, former smokers, nondaily smokers, moderate smokers, and heavy smokers. The authors found that overall diet quality between never smokers, former smokers, and nondaily smokers are largely equivalent. Conversely, compared to never smokers, moderate and heavy smokers were less compliant with dietary recommendations and heavy smokers reported less diversity in food choices; the overall ED score was not significantly different [23]. These findings acknowledge the complex nature of dietary intake across varying levels of smoking behavior.

Much of the previous literature on diet and smoking has focused on specific food types and not indicators of diet quality that can readily translate to clinical intervention. The current study addresses this knowledge gap by evaluating the relationship between dietary ED and smoking status in US adults. To date, no studies of this nature have evaluated the relationship between dietary energy density and smoking status in a US population. The types of foods consumed influence the overall dietary energy density. As previously reported, the most commonly consumed low-ED foods by this population diet include: orange vegetables, dark green leafy vegetables, fruit, salad, rice, and pasta; the most commonly consumed high-ED foods include: potato chips, savory crackers, cookies, processed cheese, and white bread [24]. A previous meta-analysis has demonstrated the robust relationship between consumption of low-ED fruits and vegetables and lower risk for mortality, particularly CVD mortality [25]. As a marker for both diet quality and a risk factor for obesity, dietary ED is an important component of understanding disorders associated with poor diet, including CVD and cancer, particularly in a population of smokers.

## Methods

### Study population & data collection

The study was conducted using data from a national representative sample of US Adults (> 18 y) who participated in the 2013–2014 National Health and Examination Survey (NHANES). The NHANES is a large cross-sectional survey conducted by both the Centers for Disease Control (CDC) and the National Center for Health Statistics (NCHS) that monitors the health and nutrition status of the non-institutionalized US residents. During the NHANES, subjects receive a comprehensive health evaluation and answer a variety of surveys, including behavioral questionnaires about dietary habits and smoking behaviors. Participants provide written consent. Complete details about NHANES survey components, survey methodology, and sampling procedures are available from the CDC NHANES website [26]. The NHANES analytic dataset includes demographic information about participants, including age at the time of exam, education level, physical activity level (measured in MET units), race/ethnicity and socioeconomic status were all provided in the NHANES data. Socioeconomic status was quantified as a continuous variable using poverty-income ratio (PIR), or the ratio of family income to family-size specific poverty threshold.

### Assessment of smoking status

During the NHANES, individuals are asked questions related to smoking status, duration, and smoking-related behaviors. Smoking status was assessed in the home, by trained interviewers using the Computer-Assisted Personal Interviewing System (CAPI). Use of the CAPI allows for increases in reporting accuracy. Participants responded to whether they currently smoke cigarettes daily, some days, or not at all. Participants were categorized as never-smokers (individuals who have smoked <100cig/lifetime), former smokers (having smoked >100cig/lifetime but do not currently smoke), and current smokers. Current smokers are further classified as daily smokers (smoking cigarettes every day) and nondaily smokers (identifies as a smoker, but does not smoke cigarettes every day).

### Assessment of dietary intake

NHANES dietary data is collected as part of the *What We Eat In America* survey [27]. Participants provide 1 day of dietary recall data, obtained by a trained interviewer using Automated Multi-Pass Method as part of the in-person medical examination. Specific status codes were provided in the NHANES data set to indicate the quality, reliability, and completeness of the dietary data. The USDA 2013–2014 Food and Nutrient Database for Dietary Studies (FNDDS) was used to process the NHANES dietary data, including micronutrient intake. Dietary Energy Density (ED) was calculated by dividing the energy content (in kcal) by weight of food (in g) consumed, excluding all beverages. Complete details of this method of calculating energy density in a nationally representative sample have been previous described [28]. In order to account for the potentially significant contribution of beverages to the total diet, beverage ED is calculated separately and used as a covariate in statistical models. This method of energy density calculation (foods only, controlling for beverage ED) is frequently used, in particular by studies showing robust relationships between dietary ED and disease status [15, 24, 28].

### Statistical analysis

For the present analyses, we initially included all adults age 18 and older (*n* = 5535) that provided complete smoking and dietary data. Women who were pregnant, and individuals with unreliable dietary data (as indicated in the NHANES or individuals reporting consuming no foods or beverages during the 24HR) were excluded, resulting in a final analytical dataset of 5293 adults. All data were analyzed using SAS version 9.4 (SAS Institute, Cary, NC) using appropriate survey weights and procedures to account for the NHANES unequal probability sampling strategy and clustered design. Multivariable regression models were used to evaluate the relationship between smoking status and dietary energy density. All models were adjusted for age, sex, race, education, socioeconomic status (PIR, poverty:income ratio), physical activity (MET-min, standardized metabolic equivalent units), beverage energy density, and body mass index. A test for linear trend using the Wald statistic was performed by modeling smoking status as a continuous variable.

## Results

Table 1 shows the demographic characteristics of the study population. Few differences in demographic characteristics were identified. Current daily smokers tend to have a lower socioeconomic status than never smokers and former smokers. It is notable that current nondaily smokers tend to be younger, and former smokers tend to be older than either never smokers or current daily smokers. Weight status distribution was relatively similar across all smoking categories, though former smokers have a higher BMI than never smokers. Interestingly, both never smokers and current nondaily smokers were more physically active than current daily smokers and former smokers.

**Table 1:** Population Characteristics by Smoking Status

	Never Smokers		Former Smokers		Current Non-Daily Smokers		Current Daily Smokers	
	*n* = 3117		*n* = 1187		*n* = 205		*n* = 844	
	n	weighted^a^ %	n	weighted %	n	weighted %	n	weighted %
Sex								
Male	1295	55.0	704	57.7*	122	59.3*	435	51.2
Female	1779	45.0	473	42.3	83	40.7	400	48.8
Race								
Non-Hispanic White	1158	61.3	608	74.5	76	51.4	455	69.7
Non-Hispanic Black	617	11.7	182	7.2	53	16.8	207	15.6
Mexican American	503	11.4	150	7.4	34	14.3	54	4.3
Other, inc. multiracial	799	15.6	237	10.9	42	17.6	119	10.4
Education Level								
HS or less	573	12.8	255	15.1	58	21.8	242	25.3
HS grad / GED	649	20.5	289	23.0	55	25.8	258	32.2
Some college / AA Degree	937	31.9	351	32.4	64	34.7	274	34.1
College graduate or above	913	34.8	281	29.5	28	17.7	60	8.4
Weight Status^b^								
Underweight	78	2.1	20	1.2	5	2.9	37	3.8
Normal weight	956	31.4	259	21.2	64	29.7	267	31.0
Overweight	918	31.3	424	35.3	58	30.3	265	31.1
Obese	1122	35.4	474	42.3	78	37.1	275	34.1
Age	44.5 ± 0.6		54.6 ± 0.4 ***		37.3 ± 1.2 **		43.0 ± 0.6	
Income (PIR)^c^	3.0 ± 0.1		3.1 ± 0.11		2.5 ± 0.2**		1.99 ± 0.1***	
Mean BMI^d^	28.8 ± 0.3		30.0 ± 0.3**		28.7 ± 0.6		28.4 ± 0.3	
Physical Activity (MET-Min)^e^	92 ± 5		74 ± 4 **		112 ± 12		70 ± 7**	

Smoking status defined as follows: never smokers have smoked <100cigarettes/lifetime, former smokers have smoked >100cigarettes/lifetime, but do not currently smoke; current non-daily smokers report currently smoking on some, but not all days; current daily smokers report currently smoking every day

^a^Weighted percentage indicates population percentages after application of NHANES survey weights

^b^Weight status is defined using standard CDC-cutpoints for BMI with underweight < 18.5 kg/^m2^ normal weight 18.5–24.9 kg/m^2^ overweight 25.0–29.9 kg/m^2^, obese > 30 kg/m^2^

^c^ Income is expressed as Poverty:Income ratio, adjusted for family size

^d^ Mean BMI is calculated adjusting for age, sex, and race. Physical activity (MET-Min) is presented as standardized metabolic equivalents, assessed for 1 week

Statistical significance indicated by the following designations: for *** *p* < 0.001, ** *p* < 0.01, * *p* < 0.05

**Fig. 1:**
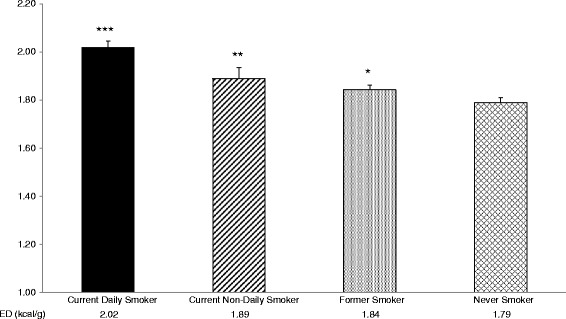
Mean dietary energy density adjusted for age, sex, race, educational attainment, socioeconomic status, BMI, beverage energy density and physical activity. Never smokers serve as the reference category.*** *p* < 0.0001 ** *p* = 0.03 *p = 0.04

Mean dietary energy density (ED, kcal/g) after adjusting for age, sex, race, educational attainment, socioeconomic status, beverage energy density, physical activity and BMI is presented in Fig. 1. Compared to never smokers, daily smokers and nondaily smokers have significantly higher dietary energy density (1.79 ± 0.02 kcal/g vs. 2.02 ± 0.03 kcal/g and 1.89 ± 0.05 kcal/g, respectively); demonstrating that any amount of current cigarette consumption is associated with poor diet. Though former smokers had a higher dietary ED (1.84 ± 0.03 kcal/g) than never smokers (*p* = 0.04), former smokers dietary ED is still significantly lower than that of current smokers (*p* = 0.002), and did not differ from nondaily smokers. The difference in energy density indicates that on average, current daily smokers consume approximately 200 cal more per day than never smokers, despite eating significantly smaller portions of food.

## Discussion

The present study assesses the relationship between smoking status and dietary behaviors. Using a nationally representative sample of US adults, we observed an inverse relationship between dietary ED and smoking status. Given the prominence of ED in national dietary guidelines and the established low intake of fruits and vegetables in smokers, dietary ED offers an effective indicator of diet quality that is amenable to intervention in smokers. Although the recent European study did not report differences in ED [23], the results of the present study highlight a similar negative correlation between smoking status and dietary quality. Both the present study and the European study evaluated smoking status using various categorizations of smokers, including former smokers. Evaluation of dietary ED among never smokers, former smokers, and current smokers indicates that former smokers have diets similar to never smokers, and better diets than current daily smokers.

The results from the present study also provide an update to the literature demonstrating that dietary behavior among smokers differs substantially from never smokers [29, 30], particularly for ED. A diet high in ED is characterized by comparatively lower consumption of fruits and vegetables. This is consistent with previous reports that smokers have lower serum Vitamin C levels and higher levels of Vitamin C turnover than non-smokers, despite an increased need for Vitamin C and other antioxidants [31]. Currently, the Recommended Dietary Allowances (RDAs) for Vitamin C for non-smokers range from 75 to 90 mg per day depending on gender, with smokers requiring an additional 35 mg per day [32], and recent reports indicate that Vitamin C intake requirements may actually be higher [33]. Increases in smoking-generated oxidative stress and decreases intakes of Vitamin C and β-carotene confer additional risk for CVD and cancer [34], presenting a major public health concern. Evaluation of specific nutrients is challenging to translate into a concrete intervention for regular smokers, particularly because recent publications indicate that antioxidant supplementation is not a recommended strategy for disease prevention [35, 36]. Therefore, understanding dietary patterns using a whole-diet approach may better allow for identification of strategies to target for intervention.

Dietary ED is a comprehensive evaluation of overall diet, as it is calculated based on both the weight and energy content of foods consumed. Both low-ED and high-ED diets have been well categorized in the literature [14, 17, 18, 24, 37]. Diets low in ED allow individuals to consume larger quantities of food for fewer calories; essentially eating satisfying portion sizes without caloric excess. Low-ED diets are also higher in diet quality [17–19, 22], and typically contain more antioxidant-rich fruits and vegetables, both of which are important factors for reduction of disease risk in smokers. Additionally, diet quality is an important predictor of post-cessation weight gain in US adults, as indicated by a 2010 analysis of data from the Framingham Heart Study Offspring Cohort [38]. Using dietary ED as a marker for diet quality may provide insight as to the rationale for the findings in the Framingham study. The findings in the present study that former smokers have diets lower in energy density than current smokers may suggest that successful cessation may be linked to prevention of weight gain, though the cross-sectional analysis does not allow for causal inferences to be determined. Consuming a diet low in energy density is a recommended strategy for preventing weight gain [16], and since concerns about weight gain may delay quit attempts [39], development of specific dietary strategies to lower dietary ED may be a valuable component of a successful smoking cessation program. Substituting higher-ED foods with low-ED, antioxidant-rich fruits and vegetables will help address micronutrient deficits associated with smoking and improve overall diet quality.

There are several strengths to the present study. Analysis of NHANES data produces results that are generalizable to the US population. The NHANES provides the unique opportunity to evaluate public health issues related to diet and smoking behaviors. The unique survey design of the NHANES allows for estimation of mean of the population’s distribution of usual dietary intake, strengthening confidence in the results presented [40]. In addition, the present study includes two distinct categories of smokers: nondaily smokers and daily smokers. Inclusion of both types of smokers allows for greater identification of dietary patterns that may be potential targets for cessation intervention. Despite recent declines in daily smoking, nondaily smokers are on the rise [1] and demonstrate similar difficulty quitting as daily smokers [41, 42]. Our results demonstrate that former smokers have better quality diets than current smokers, including those who only smoke occasionally, indicating that dietary guidance may be an important component of cessation programs for all smokers. Poor diet in smokers is consistent with other negative health behaviors often associated with smoking including sedentary behavior [43], alcohol consumption [44], and poor physical fitness [45]. The study also presents some limitations. First, dietary data is based on 24-h recall, and may be subject to bias. However, dietary data in the NHANES is collected using the USDA’s Automated Multiple-Pass Method (AMPM) with quality control procedures in place during the data collection phase can address this potential concern [40]. Finally, the cross-sectional nature of the NHANES allows for evaluation of correlation, but not causality.

## Conclusions

Our findings demonstrate an association between dietary energy and smoking status. Despite reports that, compared to never smokers, smokers have lower prevalence of obesity; however, our results demonstrate a negative linear relationship between smoking status and dietary ED. The results also demonstrate that though former smokers have a higher dietary ED than never smokers, they have better diets than current smokers, including those that only smoke occasionally. As dietary ED is a marker for both diet quality and risk factor weight-related problems, consumption of a diet low in ED may be a successful strategy for preventing weight gain following smoking cessation. Dietary recommendations based on ED are easy to implement and permit consumption of a variety of foods that are amenable to multiple personal and cultural preferences. In order to lower dietary ED, individuals must consume a greater proportion of low-ED foods, including fruits, vegetables, soups, and rice while reducing consumption of high-ED foods such as salty snacks and sweets. Diets lower in energy density contain larger portions of food for fewer calories, and are associated with higher levels of post-meal satiety [46]. Interventions that educate smokers about dietary ED in order to enable them to make educated dietary choices may help diminish concerns about weight gain as a barrier to quitting. Clinicians and researchers should do more to evaluate associations between diet quality and smoking behavior and integrate nutritional education/assessment into cessation programs.

## Abbreviations

AMPM: Automated Multiple-Pass Method
BMI: Body mass index
CAPI: Computer-Assisted Personal Interviewing System
CDC: Centers for disease control, USA
CVD: Cardiovascular disease
ED: Energy density (energy per weight of food, kcal/g)
FNDDS: USDA food and nutrient database for dietary studies
NCHS: National Center for Health Statistics
NHANES: National Health and Nutrition Examinations Surveys
ORISCAV-LUX: Observation of cardivascular risk factors in luxemburg survey
PIR: Poverty-income ratio
RDAs: Recommended daily allowances
